# Maintaining genetic diversity using molecular coancestry: the effect of marker density and effective population size

**DOI:** 10.1186/1297-9686-45-38

**Published:** 2013-10-02

**Authors:** Fernando Gómez-Romano, Beatriz Villanueva, María Ángeles Rodríguez de Cara, Jesús Fernández

**Affiliations:** 1 Departamento de Mejora Genética Animal, INIA, Ctra. de La Coruña, km. 7.5, 28040, Madrid, Spain

## Abstract

**Background:**

The most efficient method to maintain genetic diversity in populations under conservation programmes is to optimize, for each potential parent, the number of offspring left to the next generation by minimizing the global coancestry. Coancestry is usually calculated from genealogical data but molecular markers can be used to replace genealogical coancestry with molecular coancestry. Recent studies showed that optimizing contributions based on coancestry calculated from a large number of SNP markers can maintain higher levels of diversity than optimizing contributions based on genealogical data. In this study, we investigated how SNP density and effective population size impact the use of molecular coancestry to maintain diversity.

**Results:**

At low SNP densities, the genetic diversity maintained using genealogical coancestry for optimization was higher than that maintained using molecular coancestry. The performance of molecular coancestry improved with increasing marker density, and, for the scenarios evaluated, it was as efficient as genealogical coancestry if SNP density reached at least 3 times the effective population size.

However, increasing SNP density resulted in reduced returns in terms of maintained diversity. While a benefit of 12% was achieved when marker density increased from 10 to 100 SNP/Morgan, the benefit was only 2% when it increased from 100 to 500 SNP/Morgan.

**Conclusions:**

The marker density of most SNP chips already available for farm animals is sufficient for molecular coancestry to outperform genealogical coancestry in conservation programmes aimed at maintaining genetic diversity. For the purpose of effectively maintaining genetic diversity, a marker density of around 500 SNPs/Morgan can be considered as the most cost effective density when developing SNP chips for new species. Since the costs to develop SNP chips are decreasing, chips with 500 SNPs/Morgan should become available in a short-term horizon for non domestic species.

## Background

With the growing availability of genomic tools, animal genetic studies are evolving with a wide and increasing diversity of applications. In recent years, genome-wide markers have been increasingly used in selection programmes of farm animals [1] but much less in conservation programmes. One straightforward application of genomic tools in such programmes is to use information from single nucleotide polymorphism (SNP) panels to increase the accuracy of estimated genetic relationships between individuals [2,3] which would improve the strategies aimed at managing genetic diversity.

Management of populations under conservation programmes are usually aimed at maintaining the maximum possible genetic diversity (usually measured as expected and observed heterozygosity and sometimes also as allelic diversity) and avoiding high levels of inbreeding. This can be achieved by optimizing contributions of potential parents through the minimization of their global coancestry [4-6]. With a limited number of microsatellite-type markers, Fernández et al. [7] concluded that the exclusive use of molecular information to compute coancestry coefficients for the optimization process was of limited value to maintain genetic diversity compared to genealogical information. However, recently de Cara et al. [8] showed that with high-density panels of markers, the expected and observed heterozygosities maintained were higher using molecular coancestry than genealogical coancestry.

The benefits of using marker information to maintain diversity at ungenotyped loci across the whole genome depend on the amount of linkage disequilibrium (LD) between these loci and the markers used to manage the population, which itself depends on effective population size (*N*_*e*_) and marker density (*d*). In endangered populations, *N*_*e*_ is usually low since many generations and, therefore, LD is expected to be high. This enhances the potential benefits of molecular approaches to maintain genetic diversity. However, the density of available SNP panels differs largely among species [9]. While high-density panels containing tens or hundreds of thousands of SNPs have been developed in the last years for farm animal species (e.g. cattle, sheep, swine, chicken, horse and salmon), this is not the case for other species for which the payoff for SNP panels is more limited. However, as the technology becomes cheaper, arrays will be developed for non-commercial species [10]. Therefore, it is essential to determine the order of magnitude of the minimum SNP density required to maintain a significant percentage of the existing diversity through population management [9].

The aims of this study were to (i) investigate, through computer simulations, how *N*_*e*_ and SNP density affect the performance of molecular coancestry to maintain genetic diversity when used in the optimization of contributions; and (ii) determine the minimum SNP density required to maintain at least the same genetic diversity with molecular coancestry than with genealogical coancestry.

## Methods

Populations at mutation-drift equilibrium with LD between loci were generated through computer simulations. These populations were subsequently managed over ten generations based on genealogical or molecular information (see below). A large number of scenarios with different population sizes and numbers of markers per chromosome were considered.

### Generation of the base population

In order to generate a base population at mutation-drift equilibrium, 5000 discrete generations of random mating were simulated. Four different population sizes (*N*_*e*_ = 20, 40, 80 or 160 individuals, half of each sex) were considered. Sires and dams were sampled with replacement and *N*_*e*_ was kept constant across generations. Note that under this regime *N*_*e*_ equals census size (*N*). The genome was composed of 20 chromosomes of 1 Morgan each. Two types of biallelic loci (marker and non-marker loci) were simulated. Marker loci were used for management (see below) and non-marker loci were used for measuring diversity. The number of non-marker loci per chromosome was always 1000 but different densities were considered for marker loci (*d* = 10, 30, 50, 100, 500, 1000 and 2000 SNPs per chromosome). All loci were equidistant and marker loci were interspersed between the non-marker loci in such a way that they covered the whole chromosome evenly. All loci were fixed for allele 1 at the initial generation (*t* = –5000). The mutation rate per locus and generation was *μ* = 2.5 × 10^-3^ for both types of loci. The number of new mutations simulated at every generation was sampled from a Poisson distribution with mean 2*N*_*e*_*n*_*c*_*μn*_*l*_ where *n*_*c*_ is the number of chromosomes and *n*_*l*_ is the total number of loci (markers and non-markers) per chromosome. Mutations were then randomly distributed across individuals, chromosomes and loci and they switched allele 1 to allele 2. If a mutation occurred at a position where a previous mutation had already occurred, this allele was allowed to return to its previous state (i.e., 1) instead of choosing another position for the mutation but this rate of reverse mutation was very low. Individuals were mated at random. When generating the gametes, the number of crossovers per chromosome was drawn from a Poisson distribution with a mean equal to 1. Crossovers were randomly distributed without interference. For all scenarios considered, the population reached mutation-drift equilibrium after 5000 generations. We assessed this equilibrium by checking that the mean heterozygosity measured at non-marker loci was stabilized. The population at this point is referred to as the base population (*t* = 0).

In order to check the generality of the results for different mutation rates when creating the base population, some additional scenarios were run assuming a lower mutation rate (2.5 × 10^-5^). In these scenarios, *N*_*e*_ was set to 1000 and tested marker densities were *d* = 2500, 3000 and 3500 SNPs/Morgan. High values were chosen for *N*_*e*_ and *d* to achieve a reasonable number of segregating loci at *t* = 0 given this lower mutation rate.

### Management

Management of the population was carried out for ten discrete generations. Population size was kept constant across generations and equal to its size at *t* = 0 (i.e., *N* = 20, 40, 80 or 160 individuals for the high mutation rate scenarios and 1000 for the low mutation rate scenarios). The management method followed the strategy of minimizing global coancestry. Thus, the contribution of each individual (i.e., the number of offspring that each individual leaves for the next generation) was optimized by minimizing the following expression:

$$\sum_{i=1}^{N}\sum_{j=1}^{N}\frac{c_{i}c_{j}f_{\mathrm{ij}}}{{\left(2N\right)}^{2}}$$

where *c*_*i*_ is the contribution of individual *i* and *f*_*ij*_ is the coancestry between individuals *i* and *j*. Optimization was subjected to the following restrictions: both the sum of contributions of females and sum of contributions of males were equal to *N* (i.e., $\sum_{i=1}^{N_{f}}c_{i}=\sum_{i=1}^{N_{m}}c_{j}=N$, where *N*_*f*_ and *N*_*m*_ are the numbers of female and male candidates, respectively) [8]. Coancestry coefficients (*f*_*ij*_) were calculated either from molecular or genealogical data. The molecular coancestry coefficient between individuals *i* and *j* was computed as $f_{\mathrm{ij}}=\frac{1}{L}\sum_{l=1}^{L}\left[\left(\sum_{k=1}^{2}\sum_{m=1}^{2}I_{lk_{\left(i\right)}m_{\left(j\right)}}\right)/4\right]$, where *L* is the number of SNPs and $I_{lk_{\left(i\right)}m_{\left(j\right)}}$ is the identity of the *k*^*th*^ allele of individual *i* with the *m*^*th*^ allele of individual *j* for SNP *l* and takes the value of 1 if both alleles are identical and zero if they are not [11]. Molecular coancestry coefficients used in the optimization across generations were calculated using only the marker loci segregating at *t* = 0. Genealogical coancestries were calculated assuming that individuals at *t* = 0 were unrelated and not inbred. All optimizations were performed using a “simulated annealing” algorithm as described by Fernández and Toro [12].

In addition, an extra set of simulations was carried out in which genotypes for non-marker loci (i.e., loci targeted to minimize the loss of diversity) were assumed to be known and used in the optimization. These extra simulations provided the upper limit of the diversity level that could be maintained using molecular information. In these scenarios (NM), molecular coancestry coefficients were calculated from the non-marker loci and thus, management was based on the same loci that were used to measure diversity. In all simulated scenarios, once the contributions were fixed, matings between individuals were performed at random.

### Measured variables

Expected (EH) and observed heterozygosities (OH) and allelic diversity (AD) were measured over all non-marker loci and evaluated across the ten generations of management for each simulated scenario. For a single locus, EH (also called gene diversity) was calculated as $\mathrm{EH}=1-\sum_{i=1}^{2}p_{i}^{2}$, where *p*_*i*_ is the frequency of allele *i*, OH and AD were calculated, respectively, as the number of heterozygous individuals and the number of different alleles at the locus. These three variables were then averaged over all non-marker loci. The correlation between molecular and genealogical coancestry coefficients was also calculated across generations.

Linkage disequilibrium was measured at *t* = 0 as the average squared correlation coefficient between adjacent pairs of SNPs [13] which can be expressed as $r^{2}=\sum_{i=1}^{2}\sum_{j=1}^{2}\frac{D_{\mathrm{ij}}^{2}}{\left(1-p_{i}\right)\left(1-p_{j}\right)}$, where *p*_*i*_ is the frequency of allele *i* at the first locus, *p*_*j*_ is the frequency of allele *j* at the second locus and *D*_*ij*_ is the difference between the observed haplotype frequency and the expected frequency under linkage equilibrium (*p*_*i*_*p*_*j*_).

The results presented are averages over 50 replicates. A new base population at mutation-drift equilibrium was simulated for each replicate and the same base population was used for both management methods (genealogical and molecular).

### Ethical approval

The current study was carried out with the consent from the INIA Scientific Ethic Committee. We hereby confirm that the INIA Scientific Ethic Committee which is the named IACUC for the INIA approved this study.

## Results

**Figure 1:**
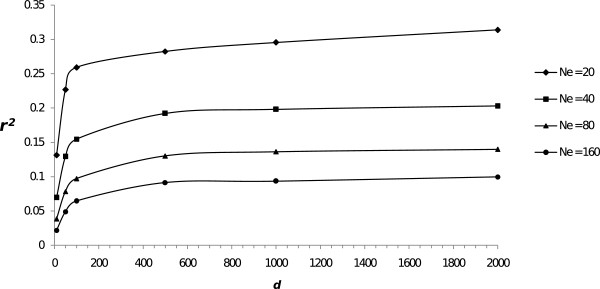
**Average linkage disequilibrium (*r***^***2***^**) between adjacent markers at the initial generation (*t*** **= 0) for different marker densities (*d*) and effective population sizes (*N***_***e***_**).**

As expected, the distribution of allelic frequencies at *t* = 0 was U-shaped. The number of fixed loci was higher in populations with a lower *N*_*e*_. The proportion of segregating markers at *t* = 0 ranged from 48 (*N*_*e*_ = 20) to 99% (*N*_*e*_ = 160). As expected, the amount of LD at *t* = 0 before management began, increased with increasing *d* and with decreasing *N*_*e*_ (Figure 1). Values for *r*^*2*^ ranged from 0.13 to 0.30 for *N*_*e*_ = 20 and from 0.02 to 0.10 for *N*_*e*_ = 160 in scenarios with the highest mutation rate. Notwithstanding, the increase observed in *r*^*2*^, when increasing *d*, was small for high densities of SNP. For the scenarios with the lowest mutation rate (*N*_*e*_ =1000), *r*^*2*^ ranged from 0.065 to 0.072. These levels of LD are in the same range as those obtained in previous studies considering similar population parameters [14,15].

Table 1 shows EH values calculated when optimization was performed with molecular or genealogical information. Results obtained using genealogical data are expressed as deviations from those obtained using molecular data. OH values (not shown) were always higher than EH values across all the scenarios simulated. The mean difference between both measures of diversity was 3% and the maximum difference, reached with the lowest initial *N*_*e*_ (*N*_*e*_ = 20) and the lowest marker density (*d* = 10 SNP/Morgan), was 8.4%. Thus, deviations from the Hardy-Weinberg equilibrium (*α* = (EH – OH)/EH) were always negative and ranged from -0.006 to -0.092. This was mainly due to sampling [16] and to a lesser extent to the management strategy implemented, which resulted in lower levels of genetic relationships than those expected using random contributions.

**Table 1:** Expected heterozygosity over generations obtained for management based on molecular or genealogical data

		***d*** **= 10**		***d*** **= 100**		***d*** **= 500**		***d*** **= 1000**		***d*** **= 2000**		**NM**^**a**^	
***N***_***e***_	***t***	**EH**_**M**_	**EH**_**M-G**_^**a**^	**EH**_**M**_	**EH**_**M-G**_	**EH**_**M**_	**EH**_**M-G**_	**EH**_**M**_	**EH**_**M-G**_	**EH**_**M**_	**EH**_**M-G**_	**EH**_**M**_	**EH**_**M-G**_
20	0	0.136	+0.000	0.136	+0.000	0.136	+0.000	0.135	+0.000	0.137	+0.000	0.136	+0.000
	1	0.131	-0.003	0.135	+0.001	0.135	+0.001	0.135	+0.001	0.137	+0.002	0.143	+0.009
	2	0.126	-0.006	0.133	+0.000	0.134	+0.001	0.134	+0.002	0.135	+0.002	0.146	+0.014
	3	0.122	-0.009	0.131	+0.000	0.132	+0.002	0.132	+0.002	0.134	+0.002	0.148	+0.017
	4	0.118	-0.011	0.129	+0.000	0.131	+0.002	0.131	+0.002	0.132	+0.003	0.149	+0.020
	10	0.100	-0.019	0.118	-0.001	0.122	+0.003	0.122	+0.004	0.124	+0.004	0.152	+0.033
160	0	0.378	+0.000	0.378	+0.000	0.378	+0.000	0.378	+0.000	0.378	+0.000	0.378	+0.000
	1	0.371	-0.007	0.377	-0.001	0.378	+0.000	0.378	+0.001	0.379	+0.001	0.390	+0.012
	2	0.366	-0.011	0.375	-0.002	0.378	+0.001	0.378	+0.001	0.379	+0.002	0.395	+0.018
	3	0.362	-0.015	0.374	-0.003	0.377	+0.001	0.378	+0.002	0.379	+0.002	0.399	+0.022
	4	0.357	-0.019	0.372	-0.004	0.377	+0.001	0.378	+0.002	0.378	+0.003	0.401	+0.025
	10	0.336	-0.036	0.364	-0.009	0.373	+0.001	0.375	+0.003	0.377	+0.005	0.413	+0.040

*EH*_*M*_ = expected heterozygosity obtained when management is based on molecular data, *EH*_*M-G*_ = expected heterozygosity obtained when management is based on genealogical data, expressed as a deviation from EH_M_, *d* = SNP density expressed in SNP/Morgan, *N*_*e*_ = effective population size, *t* = generation; ^a^molecular coancestry was calculated using the non-marker loci.

As expected, the initial heterozygosity (EH and OH) was higher in scenarios with higher *N*_*e*_ (Table 1). These scenarios also maintained a higher amount of diversity across generations than those with lower *N*_*e*_. For instance, using genealogical data the percentage of EH maintained after ten generations of management for *N*_*e*_ = 20 and *N*_*e*_ = 160 was 88% and 98%, respectively. The levels of EH at *t* = 0 ranged from 0.136 (*N*_*e*_ = 20) to 0.378 (*N*_*e*_ = 160) (Table 1). This higher value is similar to that reported by Engelsma et al. [17] who simulated a similar value of 4*N*_*e*_*μ* that is proportional to heterozygosity.

The maintained EH decreased regularly across generations in most scenarios, except when the SNP density was very high, in which case EH remained stable for several generations as previously reported and discussed by de Cara et al. [8]. In the extreme case in which non-marker loci were used in the optimization, EH even increased in the first generations.

For low densities (generally, *d* < 500), management based on genealogical coancestry resulted in higher diversity than management based on molecular coancestry and the difference in EH between these two strategies increased across generations. For instance, with *d* = 10 and management based on molecular coancestry, the EH maintained at *t* = 10 reached 74% (*N*_*e*_ = 20) and 89% (*N*_*e*_ = 160) of the initial EH but with management based on molecular coancestry it increased to 88% (*N*_*e*_ = 20) and 99% (*N*_*e*_ = 160). However, with *d* = 500, the EH maintained with management based on molecular coancestry, i.e. 90% and 99% of the initial EH for *N*_*e*_ = 20 and *N*_*e*_ = 160, respectively, exceeded that with management based on molecular coancestry, i.e. 87% and 98% for *N*_*e*_ = 20 and *N*_*e*_ = 160, respectively. The advantage of using molecular coancestries increased with increasing *d*. In any case, differences in heterozygosity between management strategies using both types of information (molecular and genealogical) were generally small.

The proportion of EH maintained at *t* = 10 using marker information in the optimization compared to non-marker information (i.e. the upper bound for EH) increased with increasing densities (Table 1). For example, with *N*_*e*_ = 20 (160), the EH maintained reached 65% (80%) of the upper bound with *d* = 10 and 77% (88%) with *d* = 100. However, with *d* > 100 the returns decreased and with *d* > 500, the increase in EH was practically negligible.

**Figure 2:**
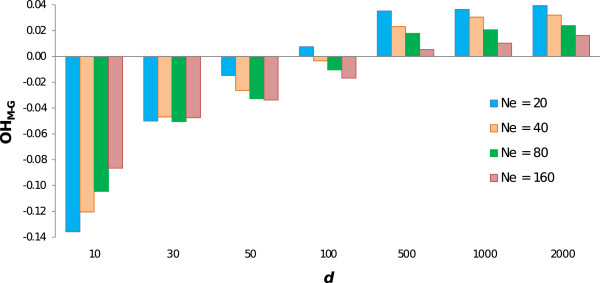
**Difference between observed heterozygosity using molecular or genealogical coancestry (OH**_**M-G**_**) at generation 10, for different marker densities (*d*) and effective population sizes (*N***_***e***_**).**

Figure 2 shows the difference in OH maintained at *t* = 10 between scenarios using molecular or genealogical coancestry for different *N*_*e*_. With a low density (e.g., *d* = 10), the performance of molecular-based management improved with increasing *N*_*e*_, although as mentioned above this performance was worse than that of genealogical-based management for all values of *N*_*e*_. With *d* > 30, the performance of molecular-based management increased with decreasing *N*_*e*_.

For the smallest *N*_*e*_ considered, a *d* = 100 (i.e., *d* = 5 *N*_*e*_ SNP/Morgan) was sufficient to reach higher levels of diversity with molecular than with genealogical coancestry. For *N*_*e*_ > 20, the density required to achieve these levels increased to 500 SNPs per chromosome. Given that scenarios with intermediate densities between *d* = 100 and *d* = 500 were not simulated, the number of markers required for management based on molecular coancestry to maintain the same levels of heterozygosity than that for management based on genealogical coancestry was estimated for each *N*_*e*_ through linear interpolation, assuming that the change in performance from *d* = 100 to *d* = 500 was constant. Values obtained were equal to about 3 times *N*_*e*_ (ranging from 2.6*N*_*e*_ to 3.3*N*_*e*_). This result showing that a SNP density of 3*N*_*e*_ SNP/Morgan for molecular coancestry was required to equalize the performance of genealogical coancestry was generalized to the scenarios in which the mutation rate used to generate the base population was reduced by two orders of magnitude (from 2.5 × 10^-3^ to 2.5 × 10^-5^) and *N*_*e*_ was increased correspondingly (*N*_*e*_ = 1000). The difference in OH maintained at *t* = 10 between scenarios using molecular or genealogical coancestry was -0.003, 0.000 and 0.002 for *d* = 2500 (*d*/*N*_*e*_ = 2.5), 3000 (*d*/*N*_*e*_ = 3.0) and 3500 (*d*/*N*_*e*_ = 3.5), respectively. Thus, the same result is found with different combinations of *μ* and *N*_*e*_.

**Figure 3:**
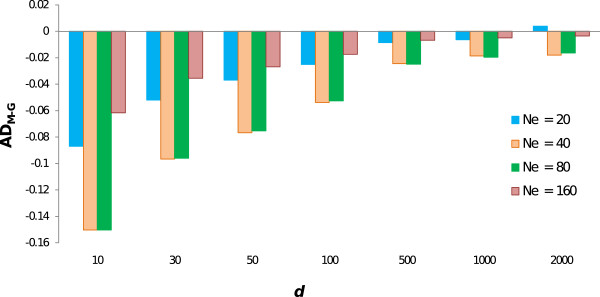
**Difference between allelic diversity using molecular or genealogical coancestry (AD**_**M-G**_**) at generation 10, for different marker densities (*d*) and effective population sizes (*N***_***e***_**).**

When AD was used as measure of diversity, genealogical coancestry was always more efficient in maintaining diversity than molecular coancestry except for the scenario with the smallest *N*_*e*_ and the highest SNP density (Figure 3). It is interesting to note that for a given *d*, the largest difference in AD between both management strategies (i.e., using genealogical or molecular coancestry) occurred at intermediate values of *N*_*e*_.

Table 2 shows the evolution across generations of the correlation between molecular and genealogical coancestries obtained when management was based on molecular data. The correlation was highest with the smallest *N*_*e*_ and the highest *d*. In general, the correlation was high (over 0.8) except in early generations for scenarios with a low *d*. Counter-intuitively, higher correlations between molecular and genealogical coancestry at *t* = 0 did not lead to smaller differences between both management methods.

**Table 2:** Correlation between molecular and genealogical coancestries

		***N***_***e***_			
***d***	***t***	**20**	**40**	**80**	**160**
10	0	0.715	0.678	0.638	0.561
	1	0.832	0.833	0.826	0.790
	5	0.859	0.864	0.846	0.804
	10	0.863	0.869	0.854	0.797
100	0	0.829	0.822	0.814	0.799
	1	0.935	0.936	0.934	0.930
	5	0.954	0.953	0.947	0.937
	10	0.954	0.953	0.947	0.935
500	0	0.848	0.837	0.835	0.832
	1	0.950	0.949	0.949	0.948
	5	0.971	0.968	0.967	0.964
	10	0.970	0.969	0.966	0.963
1000	0	0.849	0.845	0.837	0.836
	1	0.951	0.951	0.951	0.951
	5	0.973	0.971	0.970	0.968
	10	0.971	0.971	0.970	0.968
2000	0	0.848	0.848	0.841	0.838
	1	0.952	0.951	0.953	0.936
	5	0.974	0.972	0.971	0.962
	10	0.973	0.972	0.971	0.964

*d* = SNP density expressed in SNP/Morgan, *N*_*e*_*=* effective population size, *t =* generation.

## Discussion

This study investigated the effect of effective population size (*N*_*e*_) and marker density (*d*) on the efficiency of molecular coancestry when used in the optimization of contributions aimed at minimizing the loss of genetic diversity. As expected, higher densities and lower *N*_*e*_ improved the performance of the management based on molecular coancestry. This was due to the higher LD created between marker loci and non-genotyped loci at which diversity was measured. The density of SNPs required to maintain at least the same heterozygosity than that maintained using genealogical data was approximately 3*N*_*e*_ SNP/Morgan. The benefits of using molecular coancestry calculated with dense marker data were small when compared to genealogical coancestry (a benefit of 3% in the most favorable molecular scenario). However, these results differed significantly from those obtained using microsatellites [2,8] in which case management based on genealogical coancestry always outperformed that based on molecular coancestry. The combined use of genealogical and molecular information could increase furthermore the precision of the coancestry coefficient and therefore its efficiency [7].

Molecular coancestry coefficients have been calculated as the proportion of shared alleles between individuals. Many corrections aimed at making molecular coancestry closer to genealogical coancestry have been proposed and all assume that initial allelic frequencies are known [18,19]. However, at least in our context of management aimed at maintaining the highest levels of diversity, there is no advantage in applying these corrections. De Cara et al. [8] showed that when the number of markers is sufficiently large, the use of molecular coancestry always maintains higher levels of diversity than genealogical coancestry. They also tested two estimators, which did not improve the performance of molecular coancestry.

Clearly, density requirements depend on the purpose for which markers are used. In the context of genomic selection, Solberg et al. [14] reported that the accuracy of selection continued to increase with increasing marker density at least up to 8*N*_*e*_, for scenarios with *N*_*e*_ = 100. However, the increase in SNP density had reduced returns in terms of accuracy. They showed that by doubling marker density from 1*N*_*e*_ to 2*N*_*e*_ the accuracy of estimated breeding values increased by 14%. This figure was reduced to only 2% when marker density was doubled from 4*N*_*e*_ to 8*N*_*e*_. This very small increase in accuracy appears to be insufficient to justify the increase in marker density, especially taking into account that with a density of 4*N*_*e*_ SNP/Morgan the accuracy had already reached 92% of the upper bound that could be obtained theoretically [14]. Similarly, in the context of conservation programmes, the increase in SNP density had reduced returns in terms of maintained diversity (Figure 2). In scenarios with *d* = 100, the EH maintained after 10 generations ranged from 77% (*N*_*e*_ = 20) to 88% (*N*_*e*_ = 160) of the upper bound (obtained when non-marker loci were used in the optimization). These figures increased, respectively, to 80 and 90% when marker density was increased to 500 and then stayed practically constant with higher densities. Thus, under the conditions studied here, a density of 500 could be considered as the most cost effective density, given that it makes it possible to maintain a substantial amount of heterozygosity with a relatively small number of markers. Most of the SNP chips already available for farm animals (e.g. cattle, sheep, swine, chicken, horse and salmon) contain more than 500 SNP/Morgan and thus, they would be suitable for programmes on the conservation of genetic resources using a method based on the minimization of coancestry. Thus, when developing SNP chips for a new species with this objective, the marker density should reach 500 SNP/Morgan. Since the costs of developing SNP chips are decreasing, SNP chips with such densities should be feasible in a short-term horizon.

Solberg et al. [14] concluded that a density of 800 was not sufficient to achieve the maximum accuracy of genomic breeding values. This density is considerably higher than that recommended here for the maintenance of diversity (*d* = 500). For other tasks associated with conservation genetic programmes such as the determination of relatedness between individuals, a density lower than 500 SNP/Morgan would be sufficient [2].

As mentioned above, the performance of management based on molecular coancestry relative to that based on genealogical coancestry increased with decreasing *N*_*e*_, except when the density of SNPs was very low. This could be due to the fact that with a very low density, the level of LD between markers and non-genotyped loci is low even for the smallest *N*_*e*_. However, larger sample sizes (i.e., larger *N*_*e*_) can make the detection of groups of individuals with higher levels of genetic diversity possible. We observed that as *d* increased, the effect of *N*_*e*_ over the existing LD became more pronounced. The overall effect is that higher *N*_*e*_ lead to a substantial reduction in LD counteracting the beneficial effect of larger sampling sizes on the performance of management based on molecular coancestry.

Allelic diversity has been considered as an alternative measure of genetic diversity, particularly from a long-term perspective because the limits to response to selection are determined by the initial number of alleles and because allelic diversity is more sensitive to bottlenecks than EH and therefore reflects better past fluctuations in population size [20]. It should be noted that the optimization method used here was originally developed to maximize expected heterozygosity and thus AD is maintained only indirectly [21,22]. Consequently, the power of using markers to maintain AD is lower than that to maintain heterozygosity and therefore larger densities are required for molecular information to outperform genealogical management in terms of AD. In fact, this only occurred with *d* = 2000 and for *N*_*e*_ = 20. The fact that for any given *d*, the performance of management based on molecular coancestry was less good for intermediate *N*_*e*_ could also be the consequence of the opposite effects of increasing *N*_*e*_ (i.e. reduced LD but increased sample size).

As expected, the correlation between molecular and genealogical coancestries increased with increasing density but this was not translated in an increased similarity in the diversity maintained with both approaches. At *t* = 0, all individuals were assumed to be unrelated and, thus, genealogical coancestry was uniform across the individuals. This led to the equalization of contributions from all individuals. However, molecular coancestry varied and the optimization method could find a combination of contributions that resulted in higher levels of EH even when some of the candidates did not contribute at all (about 60% of the individuals did not yield offspring). The higher the number of markers, the higher was the variation in coancestry between individuals and the power of the method to discriminate between them. This led to a higher efficiency of molecular coancestry to maintain genetic diversity. Therefore, even with very high correlations between coancestries both methods produce different results.

Here, we investigated the benefits of using molecular SNP data to maintain the levels of global diversity of a population. Another advantage of using molecular information is the possibility of maintaining diversity at specific genome regions especially those responsible for adaptive variation. However, this could increase inbreeding and loss of diversity in the rest of the genome [23]. Thus, in this situation, it would be preferable to manage local and global diversity simultaneously by imposing restrictions on global coancestry while optimizing the local diversity maintained.

## Conclusions

In conclusion, a SNP density of 3*N*_*e*_ SNP/Morgan seems to be sufficient to maintain at least the same level of heterozygosity than that maintained with genealogical data. SNP chips of higher densities are available for farm animal species and they are expected to be soon available for wild species. Thus, molecular coancestry could become a powerful tool in the management of conservation programmes for the maintenance of genetic diversity.

## Competing interests

The authors declare that they have no competing interests.

## Authors’ contributions

JF and BV jointly conceived the design of the study. FGR, AC and JF developed the simulation programs. FGR performed the simulations and wrote the first draft of the manuscript. All authors discussed the results, made suggestions and corrections. All authors read and approved the final manuscript.
